# Analysis of circRNA expression in chicken HD11 cells in response to avian pathogenic *E.coli*

**DOI:** 10.3389/fvets.2022.1005899

**Published:** 2022-09-15

**Authors:** Hongyan Sun, Yexin Yang, Yuyi Ma, Nayin Li, Jishuang Tan, Changhua Sun, Huan Li

**Affiliations:** ^1^College of Animal Science and Technology, Yangzhou University, Yangzhou, China; ^2^Joint International Research Laboratory of Agriculture & Agri-Product Safety, Ministry of Education, Yangzhou University, Yangzhou, China; ^3^School of Biological and Chemical Engineering, Yangzhou Polytechnic College, Yangzhou University, Yangzhou, China

**Keywords:** APEC, RNA-seq, HD11 cells, circRNA, ceRNA regulation network

## Abstract

Avian pathogenic *E. coli* (APEC), one of the widespread zoonotic-pathogen, can cause a series of diseases collectively known as colibacillosis. This disease can cause thousands of million dollars economic loss each year in poultry industry and threaten to human health via meat or egg contamination. However, the detailed molecular mechanism underlying APEC infection is still not fully understood. Circular RNAs, a new type of endogenous noncoding RNA, have been demonstrated to involve in various biological processes. However, it is still not clear whether the circRNAs participate in host response against APEC infection. Herein, we utilized the high-throughput sequence technology to identify the circRNA expression profiles in APEC infected HD11 cells. A total of 49 differentially expressed (DE) circRNAs were detected in the comparison of APEC infected HD11 cells vs. wild type HD11 cells, which were involved in MAPK signaling pathway, Endocytosis, Focal adhesion, mTOR signaling pathway, and VEGF signaling pathway. Specifically, the source genes (*BRAF, PPP3CB, BCL2L13, RAB11A*, and *TSC2*) and their corresponding DE circRNAs may play a significant role in APEC infection. Moreover, based on ceRNA regulation, we constructed the circRNA-miRNA network and identified a couple of important regulatory relationship pairs related to APEC infection, including circRAB11A-gga-miR-125b-3p, circRAB11A-gga-miR-1696, and circTSC2-gga-miR-1649-5p. Results indicate that the aforementioned specific circRNAs and circRNA-miRNA network might have important role in regulating host immune response against APEC infection. This study is the first time to investigate the circRNAs expression profile and the biological function of the source genes of the identified DE circRNAs after APEC infection of chicken HD11 cells. These results would contribute to a better understanding of the molecular mechanisms in host response against APEC infection.

## Introduction

Colibacillosis, caused by the Avian pathogenic *E. coli* (APEC), is one of the widespread infection diseases causing extensive losses in the global poultry industry and threatening to human health via meat or egg contamination (1, 2). This disease occurred in birds of all ages, especially in younger birds aged 5–12 weeks as airsacculitis, perihepatitis, and pericarditis etc., (1, 3). In addition, APEC can not only perform as primary pathogens, but also secondary pathogens with predisposing factors for secondary infections (4, 5). Although use of antibiotic is the traditional efficient method to control APEC infection, APEC is hard to be eradicated due to drug-resistant mutations under the pressure of antibiotics (6–8). Furthermore, since APEC has multiple serotypes, vaccines can not work for all the strains (2, 9, 10). Therefore, host genetics may be an effective and sustainable way to address the APEC challenges.

Transcriptome analysis of tissues or cells at different conditions is pivotal for fully understanding the relevant gene regulatory networks. For example, Raza et al. have demonstrated that KLF6 has a role in regulating lipid metabolism (11). Also, Sandford et al. and Sun et al. separately used microarray and RNAseq to identify the gene expression profile of different immune tissues infected by APEC, screening a large number of immune genes and signal pathways against APEC infection (12–17). Moreover, Jia et al. discovered numerous miRNAs involved in chicken resistant to APEC infection and identified gga-miR-429 can regulate the platelet-derived growth factor (PDGF) and Wnt signaling pathways via *TMEFF2* and *SHISA2* against APEC infection (18).

In recent years, many reports have demonstrated that numerous circular RNAs (circRNAs) play important roles in response to a variety of viruses and bacteria (19–22). For example, the study of Qu et al. found that circRNAs are critical in the cellular innate antiviral response, that is up-regulated circRNA AIVR absorbs an miRNA that binds the mRNA of *CREBBP*, leading to an increase in the cellular expression of *CREBBP* and then accelerating IFNβ production (23). In addition, the evidences from experiment of Liu et al. showed that circRNAs may play important roles in *Chlamydia* infection by targeting endocytosis, MAPK, and PI3P-Akt signaling pathways (24). However, the knowledge of circRNAs on APEC infection is still unknown.

Considering the universal expression of circRNAs and their key role in regulating gene expression, we hypothesized that there would be differentially expressed (DE) circRNAs in chicken APEC infection, and these DE circRNAs would play important roles in chicken immune response *via* their host genes and/or miRNA during APEC infection.

## Materials and methods

### Ethical statement

The experiments were conducted under the approval of the Ethics Committee of Yangzhou University for Laboratory and Experimental Animals.

### Cell culture

The chicken cell line HD11 was was kindly provided by Dr. Xuming Hu (Yangzhou University). The cells were grown in RPMI1640 (Gibco, Carlsbad, CA, USA) with addition of 10% fetal bovine serum (FBS, Gibco, Carlsbad, CA, USA). The cells were cultured in a humidified incubator with 5% CO_2_ at 37°C. For infection, cells were challenged with 0.1 mL containing 1 × 10^8^ colony forming units (CFUs) of APEC O78 for 24 h.

### Library construction and circRNAs sequencing

Total RNA from wild type (WT) and avian pathogenic *E. coli* (APEC) infected HD11 cells was isolated by using TRIzol (Invitrogen, Waltham, MA, USA) according to the manufacturer's procedure. The total RNA purity, quality, and integrity were detected using the Qseq (Qseq100, Guangding, Taiwan). For circRNA sequencing, firstly, we take approximately 5 μg high quality RNA per sample as input material and use the Rnase R (Epicentre, USA) for each RNA sample to digest linear RNA. Secondly, ribosomal RNA was removed by Ribo-off rRNA depletion kit (Catalog NO. MRZG12324, Illumina, San Diego, CA, USA). The remaining RNAs were used to construct a cDNA library of circRNAs, and the average fragment size for final cDNA library was 250–300 bp. Finally, the cDNA library of circRNAs were 150 bp paired-end sequenced using an Illumina NovaSeq 6000 platform (Illumina San Diego, CA, USA).

### Primary analysis of circRNAs data

FastQC (v0.11.9) (25) was first used to evaluate the preliminary quality of raw sequences. After removing the adaptor sequences and low quality reads with quality scores less than Q20, the clean reads were mapped to the chicken reference genome (*gallus gallus 6.0*) by using STRA (v2.5.3a) (26). Two different algorithms, find_circ (27) and CIRCexplorer (28), were used to predict circRNA candidates. Only the identified circRNA candidates in both algorithms were further analyzed. The expression levels of circRNA candidates were calculated with back-splice junction reads. The characterization of circRNAs was statistically analyzed and the expression level of each identified circRNAs were calculated using spliced reads per billion mapping (SRPBM) method (29). The edgeR algorithm R package (30) was performed to examine the differentially expressed (DE) circRNAs. The cutoff of the significant DE circRNAs were the parameters of a |log_2_Fold Change|>0.58 and *p* value < 0.05.

### Functional analysis of source genes of DE circRNAs

Gene Ontology (GO) enrichment analysis (31) of the source genes of the DE circRNAs were implemented by using GOseq R package. KOBAS software (version 2.1.1) was used to analyzed the enrichment of significantly changed pathways for the source genes of DE circRNAs in KEGG database (32, 33).

### Construction of circRNA-miRNA Network

One of the main regulation modes of circRNAs is that they can interact with miRNAs to modulate the target gene expression (34). MiRanda software (35) was used to predict the miRNA binding sites of DE circRNA. The DE circRNAs were further selected for conjoint analysis with previous miRNA sequencing data to further obtain the DE circRNA-miRNA interactive network by using Cytoscape software.

### Validation of circRNAs by using RT-qPCR and sanger sequencing

Total RNAs were isolated from wile type and APEC infected HD11 cells by using TRIzol reagent (Invitrogen, Waltham, MA, USA), and 1 μg of total RNA was used as templates to synthesize complementary DNA (cDNA). A pair of divergent primers were designed using Primer 5.0 software to verify their head-to-tail splicing. The primers detail information for the six candidate DE circRNAs (2:8746306-8750639, 3:107147300-107151497, 1:61812485-61813589, 21:6349960-6361958, 10:18596448-18598792, 3:104232958-104234270) were listed in Supplementary Table S1. The amplified products were sequenced using Sanger sequencing. The sequences data of amplified products were aligned to the RNA-seq data and the chicken reference genome with DNAMAN (v 9.0) software to determine the authenticity of the location of the junction sites in the circular RNAs. Six genes (*DNAJB6, MTMR9, BCL2L13, CDC42, RAB11A*, and *ITSN2*) were also selected to confirm the reliability of RNA sequencing. *GADPH* was utilized as DE circRNAs quantification and internal control. Primer sequences are displayed in Supplementary Table S2. RT-qPCR thermal cycling conditions were as follows: denaturation for 3 min at 95°C, 40cycles of 10s at 95°C, 58°C for 30 s, and then 72°C for 30s. Relative expression of above genes were calculated using the 2^−ΔΔ*Ct*^ method. The formula of ΔΔCt is (Ct of gene in test group—Ct of *GAPDH* in test group)—(Ct of gene in control group—Ct of *GAPDH* in control group).

### Statistical analysis

Data analyses were performed with JMP software (version 15.2.1, SAS Institute). Results are expressed as the mean ± standard error. The statistical significance of differences between groups was evaluated by independent-samples *t*-tests and a *p* < 0.05 was considered statistically significant.

## Results

### Identification and characterization of circRNAs

A total of 77,283,432–84,964,948 sequence reads were generated, and each sample yielded approximately 77,617,332 clean reads (range from 75,293,620 to 82,496,206 as shown in Supplementary Table S3). After removing the deduplicated reads, we obtained an average of 55,216,482 reads. On average, the GC content of Unique Identifier (UID) reads was 52.1% (Supplementary Table S3). Moreover, 93.01–93.4% of the UID reads were found to successfully map to the chicken reference genome (*Gallus gallus 6.0*), of which 89.16–91.25% were uniquely mapped to genome (Supplementary Table S4). These results indicated the sequencing data were from chicken and we had good quality samples for sequencing.

**Figure 1:**
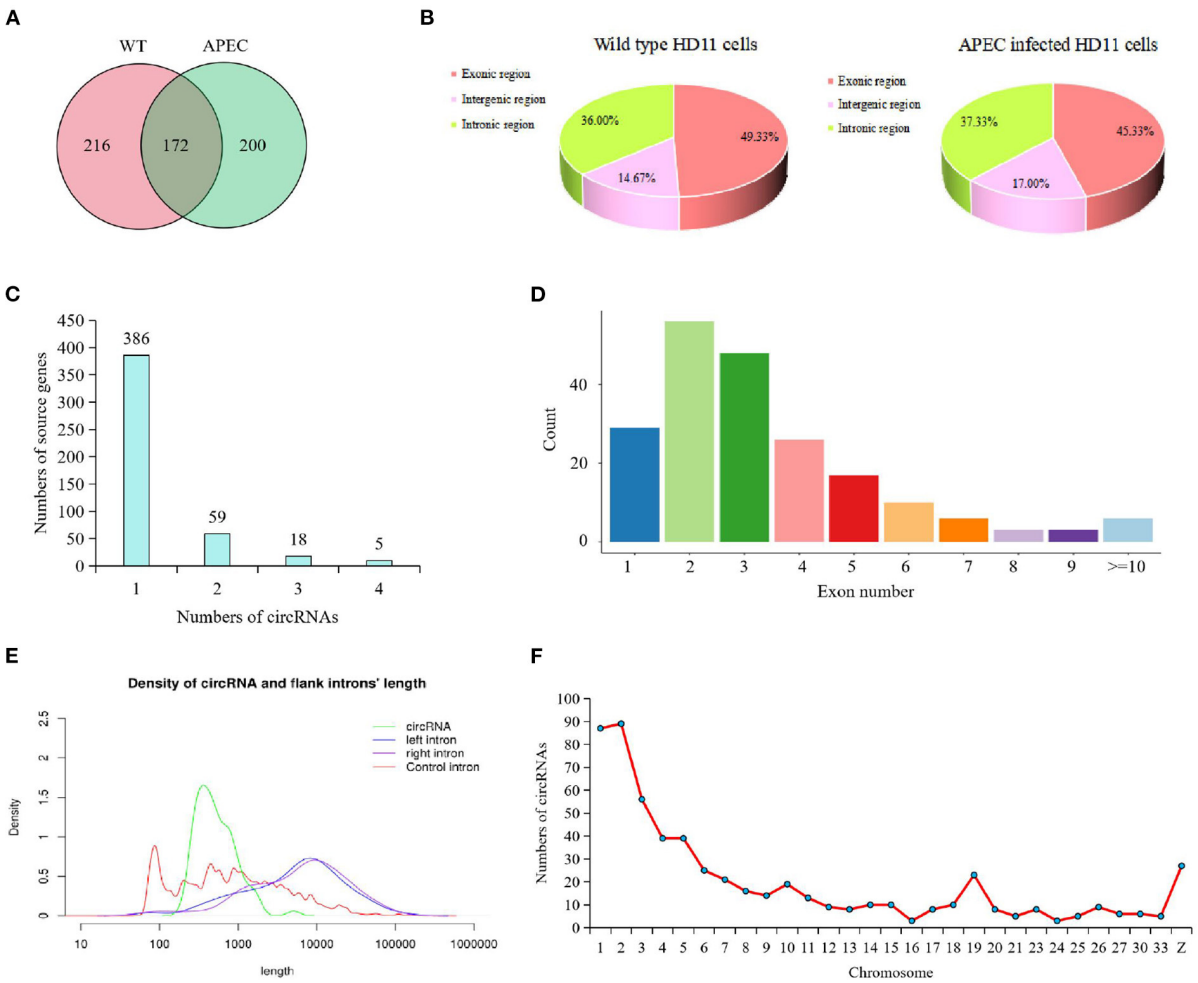
Characterization of circRNAs identified in chicken HD11 cells. **(A)** Venn diagram presenting the amount of circRNAs in wild type HD11 cells (WT) and avian pathogenic *E. coli* infected HD11 cells (APEC). **(B)** Types of circRNAs in wild type HD11 cells and avian pathogenic *E. coli* infected HD11 cells (APEC). **(C)** The analysis on alternative circularization of the identified circRNAs for different source genes. **(D)** Distribution of amount of circRNAs derived from different number of exons. **(E)** Length range distribution of the identified circRNAs. **(F)** The distribution of circRNAs on the chromosome.

After removing the linear RNA and ribosomal RNA, a total of 588 circRNAs were detected by using both find_circ and CIRC_explore methods, of which 388 were in WT group and 372 in APEC group (Figure 1A). The overlapped circRNAs in WT and APEC were 172 (Figure 1A). Additionally, in current study, 478 genes (corresponding to the 588 circRNAs) encoded at least one circRNA. Among them, 80.75% (386/478) of genes only produced one circRNA, followed by 12.34% (59/478) with two circRNAs, 3.77% (18/478) with three circRNAs, and 1.05% (5/478) with four circRNAs (Figure 1C). These results suggested that most genes yielded one circRNA, while a fraction of genes generated multiple distinguishing circularized products.

Moreover, the majority circRNAs were composed of exons (average = 47.33%) and introns (average = 36.67%), while a small proportion of circRNAs (average = 15.83%) consisted intergenic sequences (Figure 1B). It is worth to note that, compared with the WT group, the APEC group had less exonic circRNAs and more intergenic circRNAs (Figure 1B). Moreover, most of the identified circRNAs had 2-3 exons (Figure 1D). The length of most candidate circRNAs were concentrated around 900 nt (Figure 1E). The circRNAs in the present study were extensively distributed on 1-33 autosomes and sex chromosomes, of which most circRNAs were concentrated on chromosomes 1–6, 19, and Z (Figure 1F).

### Analysis of DE circRNAs

**Figure 2:**
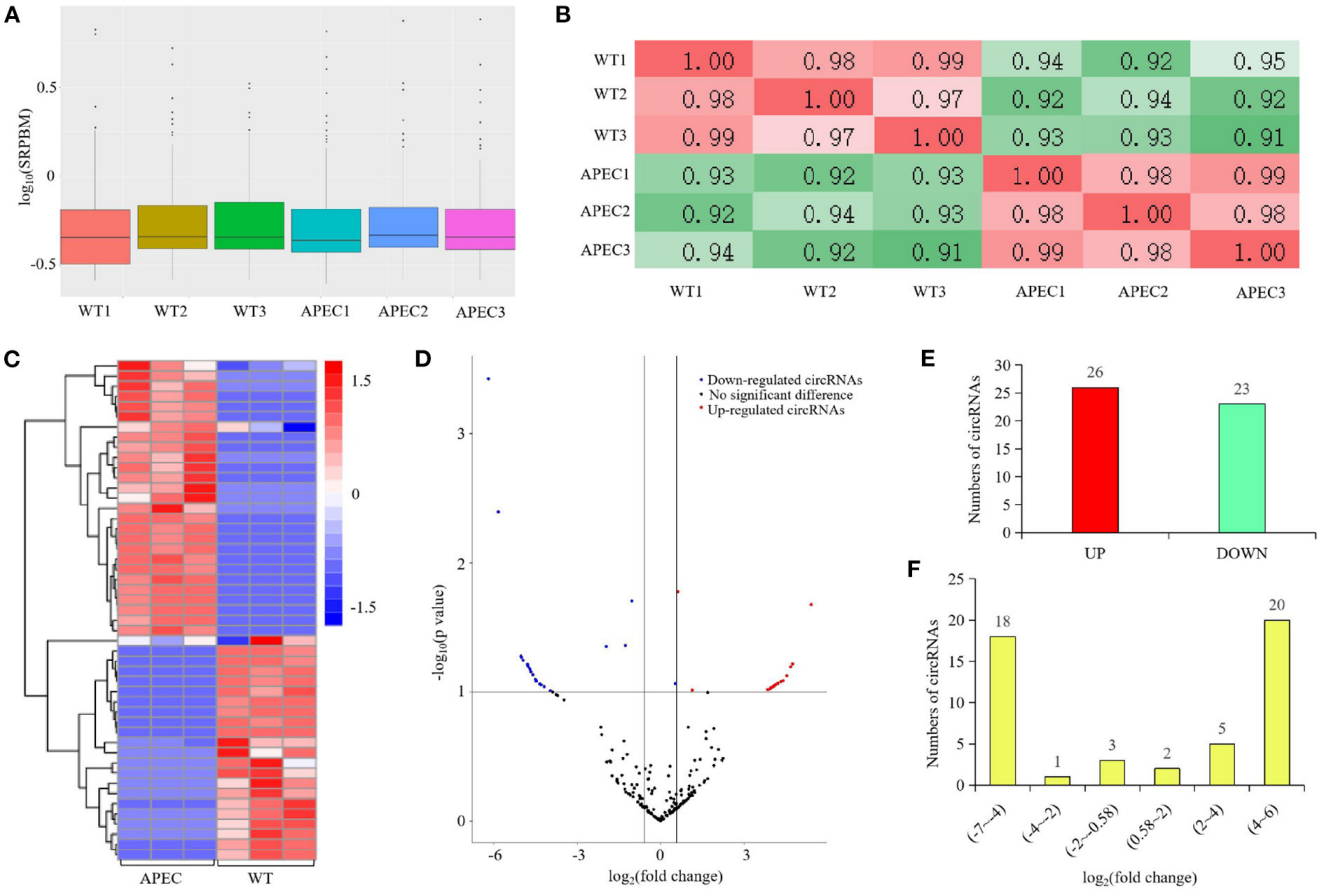
circRNA-seq profiling in the comparisons of avian pathogenic *E. coli* infected HD11 cells (APEC) vs. wild type HD11 cells (WT). **(A)** Density plot of expression distribution with density values on the vertical axis and logarithmic values of SRPBM on the horizontal axis. WT, wild type HD11 cells; APEC, avian pathogenic *E. coli*. **(B)** The heatmap of samples correlation. WT, wild type HD11 cells; APEC, avian pathogenic *E. coli*. **(C)** Heatmap analysis for the transcriptome data from the comparison of APEC vs. WT. Red color indicate up-regulation, while blue means down-regulation. **(D)** Volcano plot of circRNAs differential expression results. Red spots represent differentially expressed circRNAs for up-regulation, blue spots for down-regulation. **(E)** The expression levels of differentially expressed (DE) circRNAs in the comparison of APEC vs. WT. **(F)** The log_2_(fold change) distribution of the differentially expressed (DE) circRNAs in APEC vs. WT.

The dynamic range of the expression values was estimated and exhibited as a box plot of logarithmic transformed SRPBM (spliced reads per billion mapping) values for each sample separately (Figure 2A). Moreover, The heatmap of sample correlation showed the wild type HD11 cells group were distinct from the APEC infected HD11 cells group (Figure 2B). Meanwhile, the samples in each group had high similarity (Figure 2B). Hierarchical clustering heatmap analysis of DE circRNAs showed that the expression patterns of the circRNAs were clearly differentiated and aggregated between WT and APEC group (Figure 2C). Additionally, a total of 49 circRNAs were significantly differentially expressed in APEC vs. WT (Figure 2D), of whcih 27 DE circRNAs were identified to be up-regulated, while 22 DE circRNAs were down-regulated (Figure 2E). The log_2_(fold change) distribution of the DE circRNAs were ranged from −6.2 to 5.42, where the log_2_(fold change) of the majority circRNAs was more than 4 (Figure 2F). Among 49 DE circRNAs, the top 10 most up-regulated circRNAs and top 10 most down-regulated circRNAs are listed in Tables 1, 2.

**Table 1:** The top 10 most up-regulated circular RNAs (circRNAs) in the avian pathogenic *E. coli* infected HD11 cells compared with the wild type HD11 cells.

**circRNA ID**	**Source gene**	**Log_2_FC**	***p-*value**
4:12532765-12540210	ABCB7	5.417216504	0.021181906
4:89908603-89918509	EXOC6B	4.759295176	0.036055304
5:50719738-50729260	PPP1R13B	4.755953347	0.0461017857
2:8746306-8750639	DNAJB6	4.751193398	0.0160972907
19:8563661-8565823	AATF	4.695653868	0.0264426884
3:107147300-107151497	MTMR9	4.695653868	0.0164426884
21:3122687-3126565	ENSGALG00000002232	4.537955529	0.0075590234
6:36302501-36309401	ALDH18A1	4.401957425	0.0081659001
20:1404151-1406715	MMP24	4.398192106	0.0181745415
Z:36839947-36841327	CARNMT1	4.315241418	0.038359218

circRNA ID, circRNA in the pattern of chr:start|end; Log_2_FC, the fold change of circRNA expression.

**Table 2:** The top 10 most down-regulated circRNAs in the avian pathogenic *E. coli* infected HD11 cells compared with the wild type HD11 cells.

**circRNA ID**	**Source gene**	**Log_2_FC**	***p-*value**
5:17348967-17351158	INCENP	−4.66217647	0.006947375
4:65097900-65102412	CLOCK	−4.702116816	0.006677949
7:30699299-30701196	DARS	−4.725441341	0.016570902
10:12132977-12161781	EFL1	−4.776597946	0.036274587
7:15032338-15040086	SESTD1	−4.806089357	0.026144995
1:12744003-12750283	PTPN12	−4.943660625	0.005703412
1:56932512-56949904	BRAF	−5.012129749	0.035409144
18:4180338-4182886	MGAT5B	−5.035007979	0.015297421
10:8044919-8049376	ENSGALG00000037723	−5.851340423	0.004052171
2:120915124-120916022	ZBTB10	−6.195527687	0.00037391

circRNA ID, circRNA in the pattern of chr:start|end; Log_2_FC, the fold change of circRNA expression.

### GO and KEGG analyses of source genes of DE circRNAs

**Figure 3:**
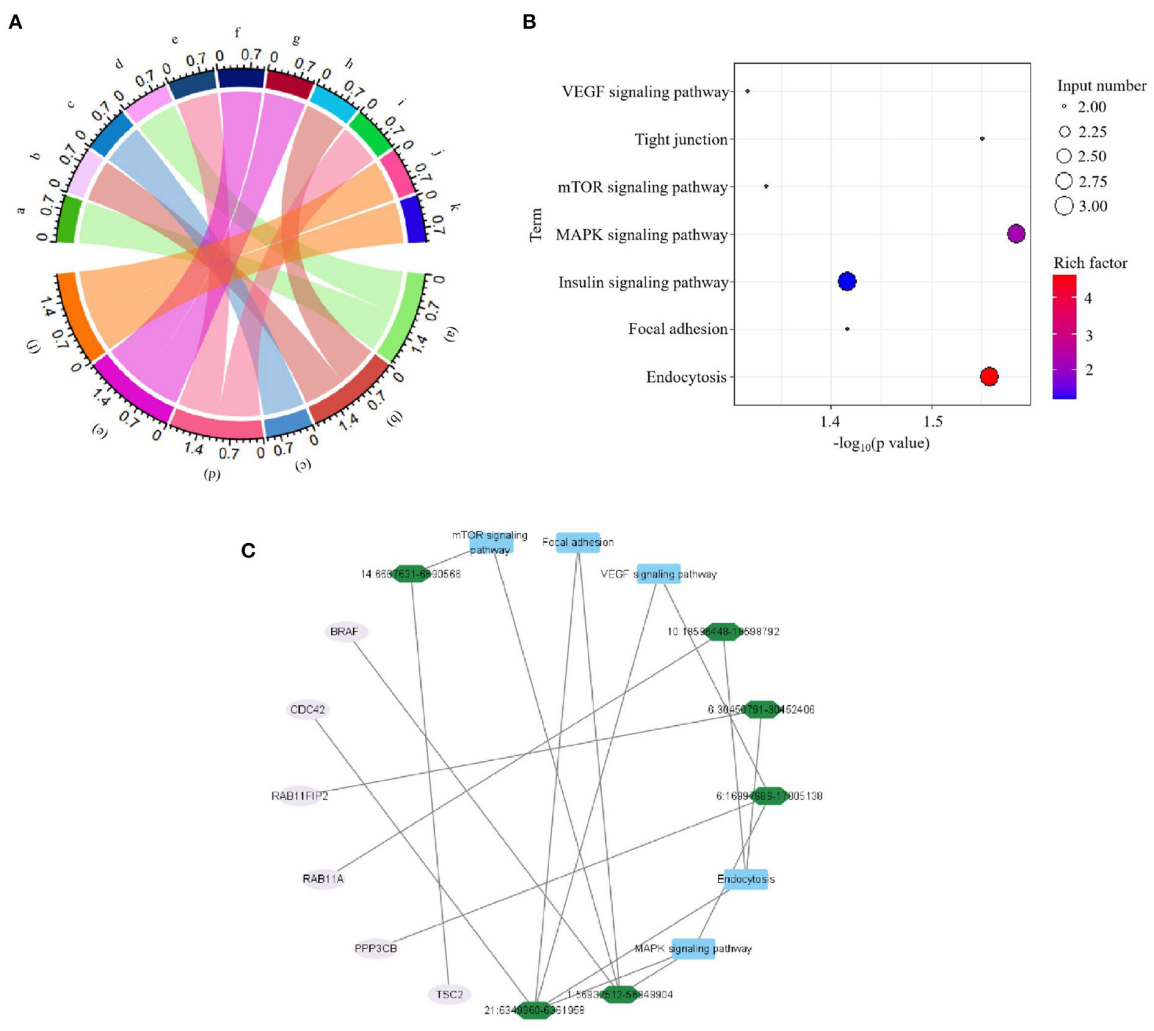
Functional analyses of source genes of differentially expressed (DE) circRNAs in the comparison of avian pathogenic *E. coli* infected HD11 cells (APEC) vs. wild type HD11 cells (WT). **(A)** Gene classification was based on Gene Ontology (GO) analysis of source genes of DE circRNAs. a. *BRAF*; b. *BCL2L13*; c. *RAB11FIP2*; d. *YBX3*; e. *CLOCK*; f. *FLOT1*; g. *ITSN2*; h. *PPP1R13B*; i. *PPP3CB*; j. *TSC2*; k. *MTMR9*; (a). positive regulation of gene expression; (b). apoptotic process; (c). phagocytosis; (d). positive regulation of inflammatory response; (e). positive regulation of endocytosis; (f). regulation of autophagy. **(B)** The significantly changed KEGG pathways in the comparison of APEC vs. WT. **(C)** Visualization for the significant enrichment pathways and enriched genes of APEC vs. WT. The innermost squares with blue represent the five significantly enriched pathways; The middle green layer represents enriched source genes; The outermost pink circles represent the identified DE circRNAs.

In current study, all the 49 DE circRNAs were derived from the exons of protein-coding genes. GO and KEGG analysis was performed to investigate the function of the DE circRNAs. It was showed that positive regulation of gene expression, apoptotic process, phagocytosis, positive regulation of inflammatory response, positive regulation of endocytosis, and regulation of autophagy biological process GO terms were detected in this experiment (Figure 3A). Meanwhile, the enriched significantly changed pathways of the DE circRNAs were MAPK signaling pathway, Endocytosis, Tight junction, Insulin signaling pathway, Focal adhesion, mTOR signaling pathway, and VEGF signaling pathway (Figure 3B). Meanwhile, the significant changed immune related pathways and their enriched source genes of DE circRNAs were visualized by using Cytoscape. As shown in Figure 3C, the result showed that six source genes (*CDC42, BRAF, PPP3CB, RAB11FIP2, RAB11A*, and *TSC2*) were detected to be enriched in the significantly changed immune related pathways.

### Verification of DE circRNAs by RT-qPCR

**Figure 4:**
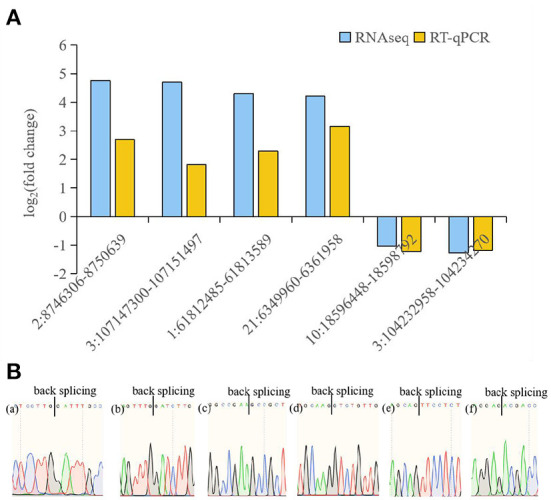
RT-qPCR analysis of circRNA expression. **(A)** RT-qPCR validation of different DE circRNAs between avain pathogenic E. coli infected HD11 cells and wild type HD11 cells. (a)-(f) represent 2:8746306-8750639 (a), 3:107147300-107151497 (b), 1:61812485-61813589 (c), 21:6349960-6361958 (d), 10:18596448-18598792 (e), 3:104232958-104234270 (f), respectively. **(B)** Sanger sequencing confirmation of the back splicing junction of circRNAs.

**Figure 5:**
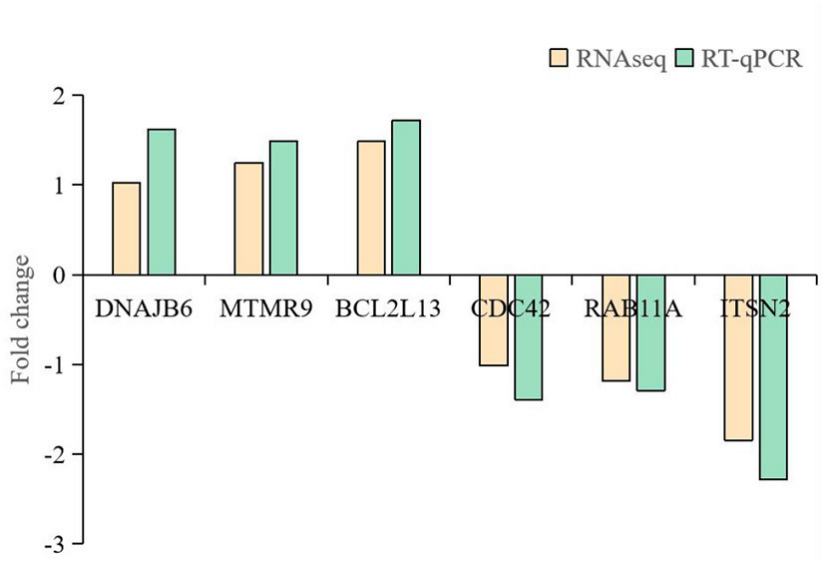
RT-qPCR analysis of mRNA expression.

Six circRNAs (2:8746306-8750639, 3:107147300-107151497, 1:61812485-61813589, 21:6349960-6361958, 10:18596448-18598792, 3:104232958-104234270) and six source genes (*DNAJB6, MTMR9, BCL2L13, CDC42, RAB11A, ITSN2*) were selected for RT-qPCR to validate the RNA-seq data. A pair of divergent primers was designed. The reverse primers of the divergent primers were located upstream of the forward primers. After confirmation by RT-qPCR, sequencing analyses were used to confirm the junction sites of the products. Results were compared with the high-throughput RNA-seq results, which showed that the expression of the six circRNAs and six source genes was consistent with the trends obtained from RNA sequencing data (Figures 4, 5).

### CircRNAs as sponges targeting miRNA

**Figure 6:**
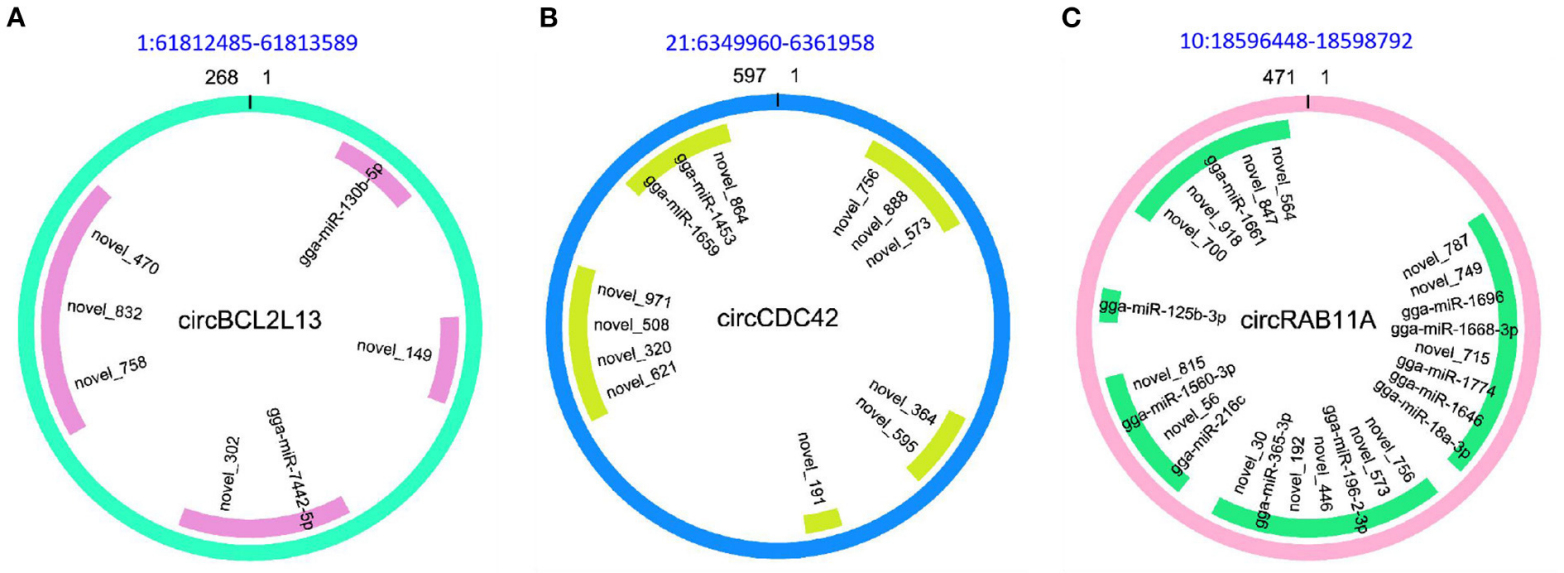
The character of sequence pairing structure between circRNA and their target miRNAs. **(A)** circBCL2L13: gga-miR-130b-5p, novel_149, gga-miR-7442-5p, novel_302, novel_758, novel_832, novel_470. **(B)** circCDC42: novel_756, novel_573, novel_888, novel_364, novel_595, novel_191, novel_621, novel_320, novel_508, novel_971, gga-miR-1659, gga-miR-1453, novel_864. **(C)** circRAB11A: novel_787, novel_749, gga-miR-1696, gga-miR-1668-3p, novel_715, gga-miR-1774, gga-miR-18a-3p, gga-miR-1646, novel_756, novel_573, gga-miR-196-2-3p, novel_446, novel_192, gga-miR-365-3p, novel_30, gga-miR-216c, novel_56, gga-miR-1560-3p, novel_815, gga-miR-125b-3p, novel_700, novel_918, gga-miR-1661, novel_847, novel_564.

Since circRNAs have the ability to affect the post-transcriptional regulation by targeting miRNA to further modulate the expression of miRNAs targets (mRNAs), the miRNA target sites of DE circRNAs in the comparison of APEC vs. WT were detected. It was found that all the 49 DE circRNAs had putative miRNA-binding sites (Supplementary Table S5). Moreover, all these circRNAs had more than one different miRNA-binding site. The largest number of miRNA-binding sites (*N* = 129) was found in circTSC2 (14:6667631-6690566). Additionally, the novel_152 can interact with the most of circRNAs (*N* = 10). Notably, the most interesting circRNAs were circBCL2L13 (1:61812485-61813589), circCDC42 (21:6349960-6361958), and circRAB11A (10:18596448-18598792) with 7, 13, and 25 miRNA-binding sites, respectively (Figure 6). These results indicate that circRNAs in chicken macrophages upon APEC infection have many potential miRNA-binding sites and probably affect the expression of immune related genes through targeting miRNA.

### Analysis of circRNA-miRNA-mRNA networks

**Figure 7:**
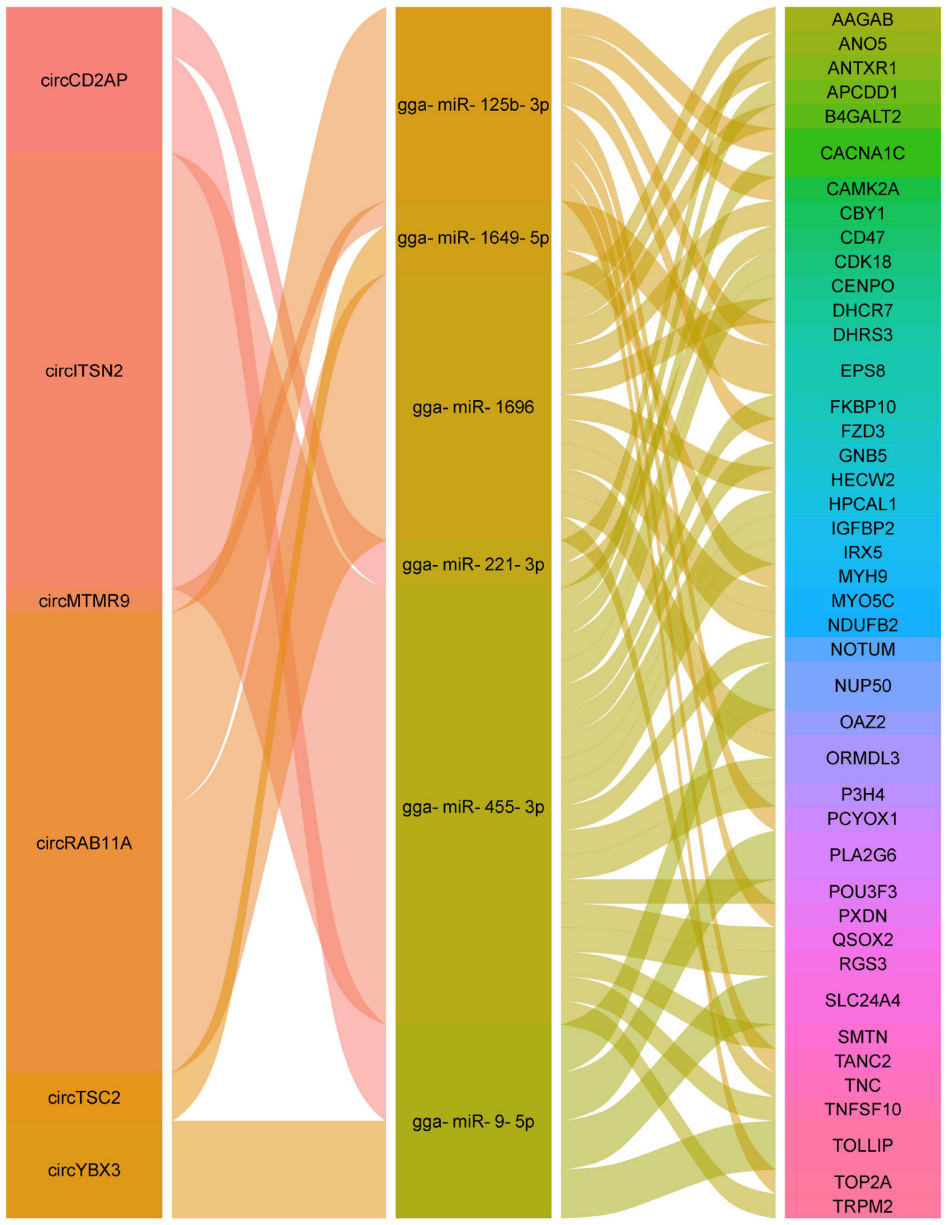
Alluvial plot of the circRNA-miRNA-mRNA interactions network.

Herein, the ceRNA regulatory networks of circRNA-miRNA-mRNA were investigated for the identified DE circRNAs by the integration of the unpublished mRNA and miRNA data. A total of 182 interactions between 18 circRNAs and 20 miRNAs were identified (Supplementary Table S6). Significantly, the four circRNAs (circCD2AP, circTSC2, circMTMR9, and circRAB11A) contained at least two potential binding sites for six miRNAs (Figure 7). Moreovr, these six miRNAs were predicted to target 43 genes whcih were significantly enriched in Wnt signaling pathway. Consequently, these results suggested that the identified circRNAs may be involved in chicken Wnt signaling pathway by sponging multiple miRNAs.

## Discussion

Although a series of experiments were performed to investigate genes expression level changes in various tissues of APEC-infected commercial broilers (12–16), the non-coding RNAs (ncRNAs), especially circular RNAs (circRNAs), remains poorly understood in response to APEC infection in chicken. CircRNAs are a large category of ncRNAs without 5' cap and a 3' poly (A) tail, generating from precursor mRNA alternative back-splicing with covalently closed continuous loop structures (29, 36). It has been demonstrated that the events of alternative circularization phenomenon (circRNAs) were found to exist widely in various species and were involved in diverse biological processes (22, 37, 38). Herein, our results suggest that significant changes occurred in the chicken transcriptome (circRNAs) upon APEC infection, which first performed the genome-wide identification and investigated the potential function of circRNAs in response to APEC infection in chicken macrophages.

As is widely known, multiple circRNAs were originated from protein-coding genes associated with alternative splicing (39–43). Up to date, a majority of researchers considered that circRNAs may function in the same pathway as the source genes (44–46), although the study of Gao et al. found that the function of circRNAs are not always in agreement with the source genes (47). Therefore, most literatures currently analyzed the KEGG and GO enrichment of the source genes to reflect the potential functions of the identified circRNAs (37, 48). In the present study, the KEGG pathway analysis identified several important pathways that mainly involved in host immune response against APEC infection, including MAPK signaling pathway, Endocytosis, Focal adhesion, mTOR signaling pathway, and VEGF signaling pathway.

Notably, an interesting exonic circRNA (1:56932512-56949904) produced by B-Raf proto-oncogene, serine/threonine kinase coding gene, *BRAF*, was identified in the comparison of APEC vs. WT in current study. It was known that *BRAF* plays an important role in regulating the MAP kinase/ERK signaling pathway to further modulate cell division, differentiation, and secretion (49–52). Moreover, mutation of *BRAF* were frequently identified in various cancers, including melanoma, non-Hodgkin lymphoma, colorectal cancer, thyroid carcinoma, non-small cell lung carcinoma (53–57). Additionally, another two most of interest circRNAs were 6:16997989-17005138 and 6:30450791-30452406, which are generated from protein phosphatase 3 catalytic subunit beta (*PPP3CB*) and RAB11 family interacting protein 2 (*RAB11FIP2*), respectively. It has been found that loss of *PPP3CB* can suppress the tumor growth *in vitro* and *in vivo* experiments (58). Moreover, the study of Skjesol et al. demonstrated that RAB11FIP2 had the ability to interact with TRAM to facilitate recruitment orchestrates actin remodeling and IRF3 activation, which are both required for phagocytosis of gram-negative bacteria (59). Consequently, it is reasonable for us to speculate that circRNAs (1:56932512-56949904, 6:16997989-17005138, and 6:30450791-30452406) may also play critical functions during APEC infection.

Furthermore, a couple of recent reports confirmed that circRNAs can function as miRNA sponges or decoys to regulate the expression of miRNA target genes (60, 61). In current study, a large number of DE circRNAs contained more than one potential miRNA binding site, indicating that these circRNAs may serve as miRNA sponges in APEC infection. For example, circRNA (1:61812485-61813589) generated from BCL2 like 13 (*BCL2L13*) gene was identified to target six miRNAs (2 known miRNA and 4 novel miRNAs). One of those circBCL2L13 targeting miRNAs, gga-miR-130b-5p, was closely related to bacteria and virus infection. The study of Fu et al. demonstrated that gga-miR-130b had the ability to suppress infectious bursal disease virus (IBDV) replication *via* targeting of the cellular suppressors of cytokine signaling 5 (SOCS5) (62). Moreover, Yuan et al. found that gga-miR-130b was involved in the defense against *Mycoplasma gallisepticum* infection of chicken via activating the PTEN/PI3K/AKT/NF-κB pathway (63). Thus, it is reasonable to speculate that circBCL2L13 might play a critical role in host defense against APEC infection by targeting gga-miR-130b-5p.

Recently, numerous studies have shown that the ceRNA networks regulation, that is the interaction among circRNAs, miRNAs, and mRNAs, was identified to widely exist in various diseases in different species (64–66). To investigate the ceRNA network and the functions of circRNAs against APEC infection in chicken macrophages, a putative circRNA-miRNA-mRNA network was constructed in current study. Of the eight regulatory relationship pairs, the three crucial pairs, circRAB11A-gga-miR-125b-3p, circRAB11A-gga-miR-1696, and circTSC2-gga-miR-1649-5p were closely related to immune response. It has been demonstrated that *RAB11A* can regulate the proliferation and motility of cancer cells via Wnt signaling pathway (67). *TSC2* was involved in modulating cellular energy response to control cell growth and survival (68). Moreover, the phosphorylated TSC2 can participate in stimulating cell growth and suppress mTOR signaling (69). Additionally, the study of Liu et al. showed that miR-125b had the ability to regulate the inflammatory response and apoptosis in *Mycobacterium tuberculosis* infected cells (70). The miR-1649 was associated with macrophage differentiation and IFNγ stimulated activation (71). MiR-1696 was involved in oxidative stress (72). Altogether, the aforementioned findings suggested that the regulatory relationship pairs of circRAB11A-gga-miR-125b-3p, circRAB11A-gga-miR-1696, and circTSC2-gga-miR-1649-5p may play important role in host immune response against APEC infection.

## Conclusion

In conclusion, the genomic characteristics, length distribution, and expression profiles of circRNAs were characterized in APEC infected macrophages and wild type macrophages. In total, 49 differentially expressed (DE) circRNAs were identified during APEC infection. Functional enrichment analysis indicated that the source genes of DE circRNAs were mainly related to MAPK signaling pathway, Endocytosis, Focal adhesion, mTOR signaling pathway, and VEGF signaling pathway. Specifically, the source genes (*BRAF, PPP3CB, BCL2L13, RAB11A*, and *TSC2*) and their corresponding DE circRNAs may play a significant role in APEC infection. Moreover, based on ceRNA regulation, we constructed the circRNA-miRNA-mRNA network and identified a couple of important regulatory relationship pairs related to APEC infection, including circRAB11A-gga-miR-125b-3p, circRAB11A-gga-miR-1696, and circTSC2-gga-miR-1649-5p. These findings will facilitate further functional research of circRNAs and lay a foundation to further understand the immune mechanism of host against APEC infection.

## Data availability statement

The datasets presented in this study can be found in online repositories. The names of the repository/repositories and accession number(s) can be found in the article/Supplementary material.

## Author contributions

HS and HL designed the experiments and wrote the original manuscript. YY and YM provided the methodology and validated the RNA-seq data. CS, NL, JT, and HL revised the manuscript. All authors have read and agreed to the published version of the manuscript.

## Funding

This research was supported by the National Natural Science Foundation of China (Grant No. 31802053), The Natural Science Foundation of Jiangsu Province (Grant No. BK20180907), the China Postdoctoral Science Foundation (2019M661950), Jiangsu Postdoctoral Science Foundation (137070510), Jiangsu Graduate Research and Practice Innovation Program (SJCX22_1796), Science and Technology Innovation Fund of Yangzhou University (X20220654), Qing Lan Project in Jiangsu Province, and Yangzhou Fourth Talent Cultivation Plan Excellent Education Talent Funding Project.

## Conflict of interest

The authors declare that the research was conducted in the absence of any commercial or financial relationships that could be construed as a potential conflict of interest.

## Publisher's note

All claims expressed in this article are solely those of the authors and do not necessarily represent those of their affiliated organizations, or those of the publisher, the editors and the reviewers. Any product that may be evaluated in this article, or claim that may be made by its manufacturer, is not guaranteed or endorsed by the publisher.
