# Supplementary material for: Analysis of circRNA expression in chicken HD11 cells in response to avian pathogenic E.coli
Source: Front Vet Sci. 2022 Sep 15;9:1005899. doi: 10.3389/fvets.2022.1005899 (PMC9521048; doi:10.3389/fvets.2022.1005899)
Supplement: Supplementary file 1 [file Table_1.DOCX]

Table S1 Specific primers for circRNAs

| circRNA ID | Forward primer(5->3) | Reverse primer(5->3) |
| --- | --- | --- |
| 2:8746306-8750639 | CCAGATGATGTCTTTAGGGAG | AACAACAGGTGGGTCTTTCA |
| 3:107147300-107151497 | TCTTATTGTCAGGGAGTGGC | CTTCTCATCATCTTTACAGCGT |
| 1:61812485-61813589 | GAAGATTGCCTGGCTCAT | GAAGCATGTTGTGCCGTA |
| 21:6349960-6361958 | ACAAGCAGAAGCCCATAA | CCATCACCCACAACTACA |
| 10:18596448-18598792 | GCGGTCCCTACAGATGAG | CCTTTATTGTCTTCCCATC |
| 3:104232958-104234270 | ACCTGGAAGTGAGAAGAAA | TCAGGCTGGAGAAATGTA |
