# Supplementary material for: Analysis of circRNA expression in chicken HD11 cells in response to avian pathogenic E.coli
Source: Front Vet Sci. 2022 Sep 15;9:1005899. doi: 10.3389/fvets.2022.1005899 (PMC9521048; doi:10.3389/fvets.2022.1005899)
Supplement: Supplementary file 2 [file Table_2.DOCX]

Table S2 Specific primers for the source genes of circRNAs

| Gene symbol | Gene ID | Forward primer(5->3) | Reverse primer(5->3) |
| --- | --- | --- | --- |
| DNAJB6 | 420448 | GCCTTACTTCGTTCTCCTC | TCTTCGTTTGCCTCACCAT |
| MTMR9 | 422033 | CTTATTGTCAGGGAGTGGC | GGCGTGCTCAAAGAGTGTA |
| BCL2L13 | 418163 | GTTCTTCACCTTATCCTCGTC | GGTTCCCTCAGTGTTTAGC |
| CDC42 | 395917 | CTCTGAAGTTGCCTTGTAT | TTTCTAAGTCTGATGGTGC |
| RAB11A | 415544 | AATCATGCTTGTGGGAAAT | GCTTCTGGGAAACAATACG |
| ITSN2 | 421979 | GAGAAACGCACTGCTAAAT | TTACAAAGGCAGCGGAACT |
| GADPH | 374193 | GTCGGAGTCAACGGATTTGG | GTTCTCAGCCTTGACAGTGCC |
