# Supplementary material for: Analysis of circRNA expression in chicken HD11 cells in response to avian pathogenic E.coli
Source: Front Vet Sci. 2022 Sep 15;9:1005899. doi: 10.3389/fvets.2022.1005899 (PMC9521048; doi:10.3389/fvets.2022.1005899)
Supplement: Supplementary file 3 [file Table_3.DOCX]

Table 3 Characteristics of RNA sequencing data

| Sample | Raw_reads | Clean_reads | Clean_GC(%) | UID_reads | UID_GC(%) |
| --- | --- | --- | --- | --- | --- |
| WT_1 | 79898294 (11.98G) | 77586290 | 51.52 | 55232248 | 51.23 |
| WT_2 | 78068340 (11.71G) | 75776170 | 52.42 | 54688864 | 52.18 |
| WT_3 | 77977654 (11.7G) | 75414976 | 52.74 | 55110018 | 52.48 |
| APEC_1 | 84964948 (12.74G) | 82496206 | 52.25 | 57401552 | 51.97 |
| APEC_2 | 77283432 (11.59G) | 75293620 | 52.72 | 53680588 | 52.48 |
| APEC_3 | 81346714 (12.2G) | 79136728 | 52.53 | 55185622 | 52.28 |
