# Supplementary material for: Analysis of circRNA expression in chicken HD11 cells in response to avian pathogenic E.coli
Source: Front Vet Sci. 2022 Sep 15;9:1005899. doi: 10.3389/fvets.2022.1005899 (PMC9521048; doi:10.3389/fvets.2022.1005899)
Supplement: Supplementary file 4 [file Table_4.DOCX]

Table 4 The reads mapping information for each sample

| Sample | Total reads | Total mapped (%) | Non-unique (%) | Unique (%) | Unmapped reads (%) |
| --- | --- | --- | --- | --- | --- |
| WT_1 | 55232248 | 51589617  (93.40) | 4512427  (8.75) | 47077190  (91.25) | 3642631  (6.60) |
| WT_2 | 54688864 | 50974341  (93.21) | 5295239  (10.39) | 45679102  (89.61) | 3714523  (6.79) |
| WT_3 | 55110018 | 51275004  (93.04) | 5560473  (10.84) | 45714531  (89.16) | 3835014  (6.96) |
| APEC_1 | 57401552 | 53393248  (93.02) | 5117638  (9.58) | 48275610  (90.42) | 4008304  (6.98) |
| APEC_2 | 53680588 | 49970523  (93.09) | 4942818  (9.89) | 45027705  (90.11) | 3710065  (6.91) |
| APEC_3 | 55185622 | 51327487  (93.01) | 4854328  (9.46) | 46473159  (90.54) | 3858135  (6.99) |
